# A Longitudinal Aging and End-of-Life Care Curriculum for Medical Students Using the Geriatric 5Ms Framework

**DOI:** 10.1093/geroni/igaa057.029

**Published:** 2020-12-16

**Authors:** Andrea Schwartz, Kristen Schaefer

**Affiliations:** 1 Harvard Medical School, VA Boston, New England GRECC, Boston, Massachusetts, United States; 2 Dana Farber Cancer Institute, Brigham & Women’s Hospital, Harvard Medical School, Boston, Massachusetts, United States

## Abstract

Medical student training in geriatrics and palliative care is critical to prepare them to care for older adults and those facing serious illness. We created a longitudinal Aging and End of Life Care Curriculum at Harvard Medical School, using Kern’s Curriculum Design Model. We conducted a focused needs assessment survey with course and clerkship directors, then implemented curricula based on the AAMC and Hartford Foundation’s 26 learning objectives in Geriatrics (Leipzig et al, Acad Med 2009), and “Raising the Bar for the care of seriously ill patients” which established competencies for medical students in palliative care (Schaefer at al, Acad Med 2014). We structured the curricular content to enable spaced learning, using the Geriatric 5Ms framework of Mobility, Mind, Medications, Multi-complexity and Matters Most (Tinetti at al, JAGS 2017), which aligns with the Age Friendly Health Systems Initiative priorities. Students participate in trainings on Delivering Serious News and Goals of Care Conversations, structured home visits with older adults, and clinical reasoning sessions focused on falls, delirium and polypharmacy risk reduction. The curriculum includes interactive, case based and jigsaw learning, as well as flipped classroom learning. Students are evaluated using a three part longitudinal Objective Structured Clinical Examination with an aging patient, which demonstrates an increase in medical student clinical skills in geriatrics. Individual sessions of the curriculum demonstrate increases in student knowledge of and attitude to geriatrics; longitudinal assessment is ongoing to ensure that students graduate ready to care for an aging society with competence, knowledge and compassion.

